# Growth and Biochemical Composition of *Porphyridium* *purpureum* SCS-02 under Different Nitrogen Concentrations

**DOI:** 10.3390/md17020124

**Published:** 2019-02-20

**Authors:** Tao Li, Jin Xu, Houbo Wu, Peiliang Jiang, Zishuo Chen, Wenzhou Xiang

**Affiliations:** 1 CAS Key Laboratory of Tropical Marine Bio-resources and Ecology, Guangdong Key Laboratory of Marine Materia Medica, Institution of South China Sea Ecology and Environmental Engineering, RNAM Center for Marine Microbiology, South China Sea Institute of Oceanology, Chinese Academy of Sciences, Guangzhou 510301, China; taoli@scsio.ac.cn (T.L.); wuhoubo@scsio.ac.cn (H.W.); 18390943716@163.com (Z.C.); 2 Key Laboratory of Renewable Energy, Guangzhou Institute of Energy Conversion, Chinese Academy of Sciences, Guangzhou 510640, China; xujin@ms.giec.ac.cn (J.X.); jiangyang9977@163.com (P.J.)

**Keywords:** *Porphyridium*, nitrogen, phycoerythrin, exopolysaccharide, polyunsaturated fatty acids

## Abstract

Microalgae of the genus *Porphyridium* show great potential for large-scale commercial cultivation, as they accumulate large quantities of B-phycoerythrin (B-PE), long chain polyunsaturated fatty acids (LC-PUFAs) and exopolysaccharide (EPS). The present study aimed to adjust culture nitrogen concentrations to produce *Porphyridium* biomass rich in B-PE, LC-PUFAs and EPS. *Porphyridium purpureum* SCS-02 was cultured in ASW culture medium with low nitrogen supply (LN, 3.5 mM), medium nitrogen supply (MN, 5.9 mM) or high nitrogen supply (HN, 17.6 mM). HN significantly enhanced the accumulation of biomass, intracellular protein, B-PE and eicosapentaenoic acid. LN increased the intracellular carbohydrate and arachidonic acid content, and promoted the secretion of EPS. The total lipids content was almost unaffected by nitrogen concentration. Based on these results, a semi-continuous two-step process was proposed, which included the production of biomass rich in B-PE and LC-PUFAs with sufficient nitrogen, and induced EPS excretion with limited nitrogen and strong light.

## 1. Introduction

*Porphyridium purpureum* is a species of marine red algae in the Porphyridiophyceae family. It has received great attention in recent years because of its potential to produce B-phycoerythrin (B-PE), long chain polyunsaturated fatty acids (LC-PUFAs) and exopolysaccharides (EPS) which are excellent feedstock for food, nutraceuticals and pharmaceuticals [1,2,3]. However, to date, large-scale cultivation and commercial application of *P*. *purpureum* has not achieved worldwide implementation. For large-scale cultivation of *P*. *purpureum*, a crucial problem is how to promote the yield of B-PE, LC-PUFAs and EPS [4]. Interestingly, many kinds of microalgae can change their biochemical composition or accumulate secondary compounds when under stress conditions, such as those of strong light intensity or nitrogen limitation [5,6]. This provides a possible approach to the production of target biochemicals by changing the culture conditions. This method has been applied in the production of astaxanthin from *Haematococcus pluvialis*, oil from oleaginous microalgae and phycocyanin from *Spirulina platensis* [6]. However, it is not clear whether the above-mentioned method can be used for the production of *P*. *purpureum* biomass that is simultaneously rich in B-PE, LC-PUFAs and EPS.

Nitrogen is an essential component of key enzymes, photosynthetic pigments and genetic materials and other nitrogen-containing compounds that are necessary to maintain microalgal growth, and limitation of nitrogen can notably curtail the growth of microalgae [7]. In particular, limitation of nitrogen can enhance the accumulation of lipids and carbohydrates, but suppress the accumulation of proteins [8]. Several studies examining the effects of nitrogen on the accumulation of B-PE, LC-PUFAs and EPS in *P*. *purpureum* have been reported in the past 10 years [9,10,11]. B-PE is one of the most well-known pigment-protein complexes; limitation of nitrogen can significantly inhibit the synthesis of B-PE, while sufficiency of nitrogen can substantially enhance the synthesis of B-PE [12]. EPS is a type of negatively charged sulphated polysaccharide, the average molecular weight of which ranges from 1.4 × 10^6^ to 1.7 × 10^6^ Da [3]. Under nitrogen-sufficient conditions, *P*. *purpureum* only secrets a small amount of EPS; but under nitrogen-limited conditions, the synthesis of EPS is promoted to some extent [1]. LC-PUFAs in *P*. *purpureum* mainly include arachidonic acid (C20:4, ARA) and eicosapentaenoic acid (C20:5, EPA) [13]. Reports indicate that most EPA was present mainly in membrane lipids but most of the ARA was found in the storage lipids [14]. Nitrogen limitation promotes the accumulation of storage lipids, but reduces the accumulation of membrane lipids [8]. However, contradictory results regarding the effect of low nitrogen concentration on EPA and ARA production have been reported in many of the previous studies [9,15,16]. These changes in B-PE, LC-PUFAs and EPS concentrations resulting from the different nitrogen concentrations provide to be a theoretical foundation for the customised production of these three compounds. In addition to nitrogen, many other factors such as light intensity, phosphate, light wavelengths, temperature and oxygen concentration have also great effect on B-PE, LC-PUFAs and EPS synthesis. Guihéneuf et al. (2015) reported that an increase in light and temperature caused a strong decrease in the content of phycobiliproteins and EPA in *P. purpureum* [12]. The highest phycobiliproteins content (2.9% DW) was reached under combined low light (30 μmol photons m^−2^ s^−1^) and low temperature (10 °C) [12]. The results from del Pilar Sánchez-Saavedra et al. (2018) showed that the high light intensity could significantly improve the content of carbohydrate, protein and B-PE content [17]. Hu et al. (2018) showed that phosphate limitation was not conducive to the accumulation of PUFAs [9]. Soanen et al. (2016) showed that the increase in light and temperature could enhance the secretion of EPS in *Porphyridium marinum* [18]. Coward et al. (2016) reported that green light could improve the content phycobiliproteins [19]. Rogova et al. (1996) showed that the content of EPA and ARA was enhanced by the high oxygen concentration [20].

Previous studies have attempted to increase the yield of B-PE, LC-PUFAs and EPS using different methods. Uniform design was used to improve EPS production by optimising renewal conditions, which included the concentration of NaNO_3_, renewal rate and renewal period. The maximum output rate of EPS was 68.64 mg L^−1^ day^−1^ [11]. Another study by Singh et al. (2000) reported that the maximal volumetric concentration of EPS (1.32 g L^−1^) produced by *Porphyridium* sp. was obtained outdoors during the winter months using flat plate glass reactors (1.3 cm) [21]. Kathiresan et al. (2007) conducted the optimum of the major constituents in culture medium using response surface methodology (RSM) to enhance the content of B-PE. Under the optimum conditions, the content of B-PE reached 3.3% DW [22]. Su et al. (2016) showed that phosphate limitation could promote LC-PUFAs content. The highest total fatty acids (TFA) yield of 392.59 mg L^−1^ and the content of 34.69 mg g^−1^ were obtained at a phosphate concentration of 0.035 g L^−1^ [16]. Fuentes-Grünewald et al. (2015) in their study showed that semi-continuous culture was the most feasible strategy to produce *P*. *purpureum* biomass; the average biomass produced using this method was 47.04 mg L^−1^ day^−1^, with an EPS production of 2.1 g L^−1^ [23]. To date, no method has yet been developed that allows *Porphyridium* to produce high yields of B-PE, LC-PUFAs and EPS simultaneously. High yields of B-PE and LC-PUFAs from *Porphyridium* would likely accumulate under optimal culture conditions, such as those of sufficient nitrogen and low light intensity. However, rapid secretion of EPS is usually achieved under stress conditions, such as those of limited nitrogen and strong light intensity.

In the present study, we report the isolation of *P*. *purpureum* SCS-02 from the South China Sea. This microalga strain has strong environmental adaptability, and can grow normally outdoors between 5 °C and 35 °C. *P*. *purpureum* SCS-02 was cultured at three initial nitrogen concentrations: low nitrogen supply (LN, 3.5 mM), nitrogen supply (MN, 5.9 mM), and high nitrogen supply (HN, 17.6 mM). During the cultivation period, 1% CO_2_ was continuously bubbled into the photobioreactor to ensure a sufficient source of carbon. The present research focused primarily on the growth, B-PE, EPS and LC-PUFAs production of *P*. *purpureum* SCS-02 under different initial nitrogen concentrations. The results will provide an optimal strategy for the customised production of B-PE, EPS and LC-PUFAs from *P*. *purpureum* SCS-02 by adjusting the initial nitrogen concentration.

## 2. Results

### 2.1. The Growth of P. purpureum SCS-02 under Different Nitrogen Concentrations

**Figure 1:**
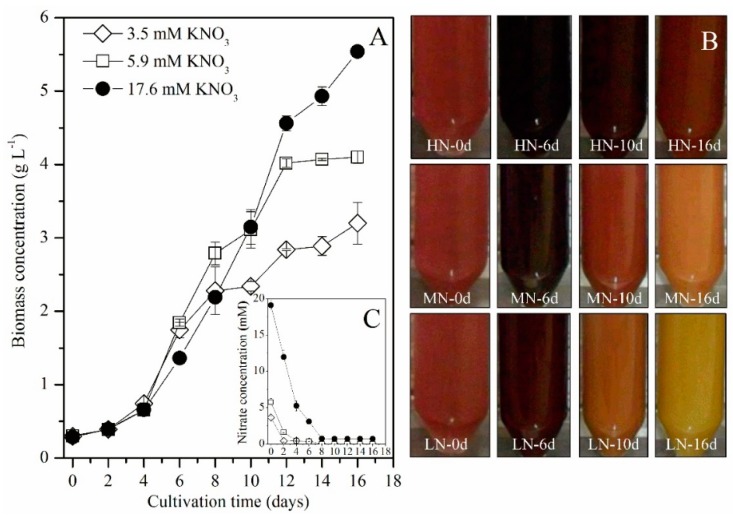
Growth characteristics of *P*. *purpureum* SCS-02 under different nitrogen concentrations. (**A**) biomass concentration; (**B**) colour change; (**C**) nitrate concentration. LH: low nitrogen supply (3.5 mM); MN: medium nitrogen supply (5.9 mM); HN: high nitrogen supply (17.6 mM). The values shown are the averages of two biological replicates and three technical replicates ± standard deviation.

Under LN, MN and HN culture conditions, *P*. *purpureum* SCS-02 showed marked differences in growth (Figure 1A). From day 0 to day 4, there were no differences in biomass concentration between any of the nitrogen concentrations. From day 6 to day 10, the increase in biomass concentration of the MN group exceeded that of the LN and HN groups. After day 10, the increase in biomass concentration of the LN and MN groups was noticeably reduced, but the biomass concentration of the HN group increased rapidly. On the final day of culture, the biomass concentration of the HN group reached 5.54 g L^−1^, which is 35% and 73% higher than that of the MN and LN groups, respectively.

The colour change of *P*. *purpureum* SCS-02 during cultivation is shown in Figure 1B. At the beginning of the culture, all treatments showed a purple-red colour. From day 0 to day 6, the colour of the culture deepened in intensity in all treatments. However, after day 6, the purple-red colour gradually faded. Furthermore, the rate at which the colour faded in the LN group was faster than that in the MN and HN groups. On the final day of culture, the colour of the LN and MN groups turned yellow and orange, respectively. The HN group maintained the purple-red colour, but the colour intensity was less than that on day 6. As can be seen from Figure 1C, the initial nitrogen concentration of LN, MN and HN groups was 3.67 mM, 5.76 mM and 19.10 mM, respectively which were similar to the theoretical data. The time point of nitrogen limitation was day 2 for LN, day 4 for MN and day 8 for HN.

### 2.2. Changes in P. purpureum SCS-02 Biochemical Composition under Different Nitrogen Concentrations

**Figure 2:**
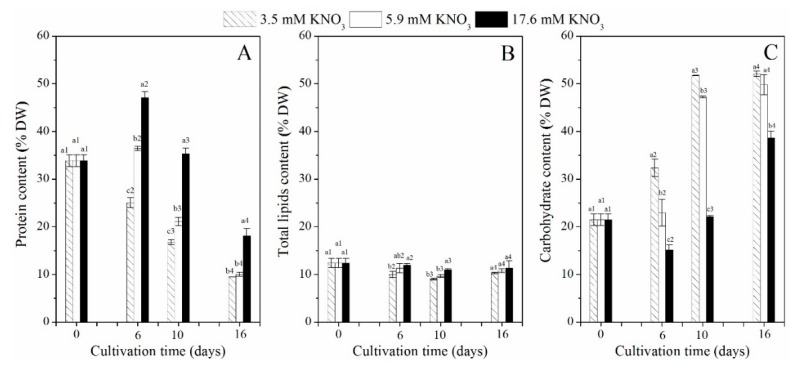
Changes of biochemical composition of *P*. *purpureum* SCS-02 under different nitrogen concentration. (**A**) protein; (**B**) total lipids; (**C**) carbohydrate. Different letters denoted significant differences among the values of the protein content, total lipids content and carbohydrate content on the different nitrogen concentration (a1–c1: day 0; a2–c2: day 6; a3–c3: day 10; a4–c4: day 16). The values shown are the averages of two biological replicates and three technical replicates ± standard deviation.

The carbohydrate and protein content of *P*. *purpureum* SCS-02 showed significant changes with the culture time and initial nitrogen concentration, while few changes in total lipid contents were observed (Figure 2A,C). The protein content of the MN and HN groups increased rapidly from day 0 to day 6, and decreased from day 6 to day 16. On day 6, the protein content of the HN group reached 47.1% DW (dry weight), which was significantly higher than the protein content of the MN group (*p* < 0.01). However, the LN group showed a decreasing trend throughout the culture period. On the final day of culture, the *P*. *purpureum* SCS-02 protein content in the LN, MN and HN groups was 9.5% DW, 10.0% DW and 18.1% DW, respectively.

As the culture progressed, the carbohydrate content revealed a gradually increasing trend in the LN, MN and HN groups, but the rate of carbohydrate accumulation was notably impaired (Figure 2C). The LN group showed a faster rate of carbohydrate accumulation did than the MN and HN groups. On day 16, the maximum carbohydrate content reached 52.1% DW in the LN group, 49.8% DW in the MN group and 38.7% DW in the HN group.

Throughout the culture period, the total lipid content exhibited less change of between 9% DW and 12% DW under LN, MN and HN conditions (Figure 2B). The total lipid content of the HN group was slightly higher than that of the LN and MN groups (*p* > 0.05).

As shown in Table 1, the protein yield under HN conditions was 1.01 g L^−1^, which was 2.37 times that of the LN group and 1.46 times that of the MN group (*p* < 0.01). The total lipid yield under HN conditions was 0.63 g L^−1^, which increased by 90.0% and 30.0% as compared with that in the LN and MN groups (*p* < 0.01), respectively. The carbohydrate yield under HN conditions was 2.14 g L^−1^, which was 28.1% higher than that of the LN group (*p* < 0.01), but was similar to that of the MN group at 2.04 g L^−1^ (*p* > 0.05). These results suggest that biomass concentration made an important contribution to the yield of carbohydrates, total lipids and proteins.

### 2.3. Changes in P. purpureum SCS-02 EPS Concentration under Different Nitrogen Concentrations

**Figure 3:**
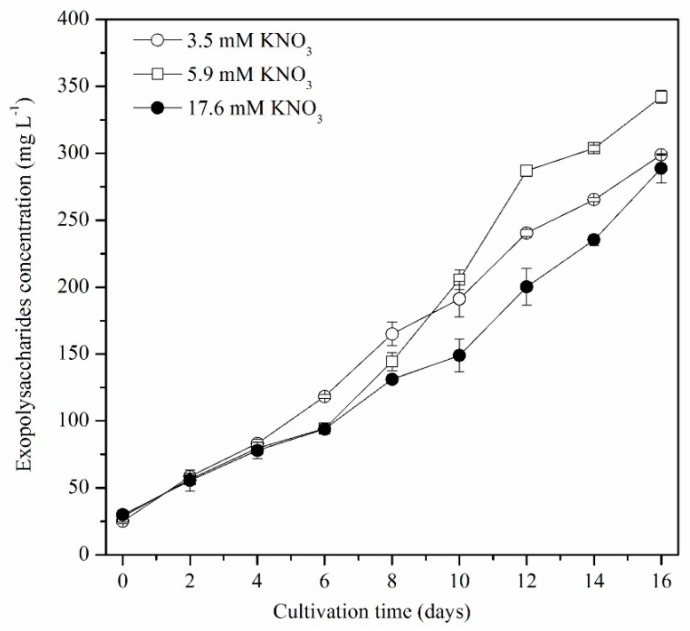
Changes in exopolysaccharide concentration of *P*. *purpureum* SCS-02 under different nitrogen concentrations. The values shown are the averages of two biological replicates and three technical replicates ± standard deviation.

*P*. *purpureum* SCS-02 secretes EPS during growth. The change in EPS concentration during each growth phase is shown in Figure 3. During culture growth, the EPS concentration showed an increasing trend under the LN, MN and HN conditions. From day 0 to day 4, there were no significant differences between treatments. From day 4 to day 10, the LN group exhibited a faster rate of EPS secretion than the MN and HN groups. After day 10, the secretion rate of EPS in the MN group exceeded the secretion rate of the HN and LN groups. By the final day of culture, the MN group achieved the highest EPS concentration (0.342 g L^−1^), which was 14.8% and 18.7% higher than the LN groups (0.299 g L^−1^) and HN groups (0.288 g L^−1^), respectively.

### 2.4. Variation in P. purpureum SCS-02 Fatty Acid Composition under Different Nitrogen Concentrations

**Figure 4:**
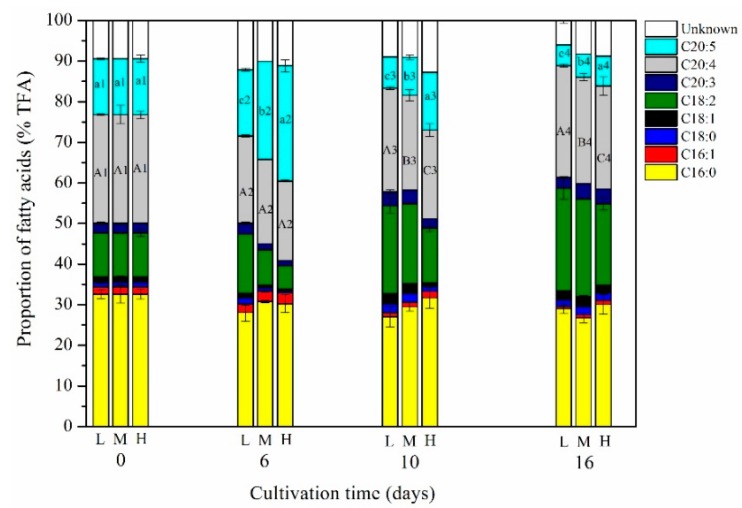
Fatty acid composition of *P*. *purpureum* SCS-02 under different nitrogen concentrations. L: Low nitrogen supply (3.5 mM); M: medium nitrogen supply (5.9 mM); H: high nitrogen supply (17.6 mM). TFA: total fatty acid. Different capital letters denoted significant differences among the values of ARA (20:4) proportion in the different nitrogen concentration (a1–c1: day 0; a2–c2: day 6; a3–c3: day 10; a4–c4: day 16). Different lowercase letters denoted significant differences among the values of EPA (20:5) proportion in the different nitrogen concentration (a1–c1: day 0; a2–c2: day 6; a3–c3: day 10; a4–c4: day 16). The values shown are the averages of two biological replicates and three technical replicates ± standard deviation.

The main fatty acids of *P*. *purpureum* SCS-02 include C16:0, C16:1, C18:0, C18:1, C18:2, C20:4 (ARA, ω-6) and C20:5 (EPA, ω-3). Among these fatty acids, few changes were observed in the proportions of C16:0, C16:1, C18:0 and C18:1 that were present, while culture time and nitrogen concentration produced apparent changes in the proportions of C18:2, ARA and EPA (Figure 4). The maximum proportion of EPA was detected on day 6, which was 16.36% TFA in the LN group, 24.14% TFA in the MN group and 28.38% TFA in the HN group. From day 6 to day 16, the proportion of EPA showed a decreasing trend. On day 16, the proportion of EPA under the LN, MN and HN groups decreased to 5.09% TFA, 5.71% TFA and 7.39% TFA, respectively. The LN conditions promoted the accumulation of ARA, which was 27.51% TFA on day 14, an increase of 8.03% over that of the HN group (*p* < 0.01). LN conditions were more beneficial to the accumulation of C18:2. On the final day of cultivation (day 16), the proportion of C18:2 under LN, MN and HN conditions was 25.26% TFA, 24.03% TFA and 20.09% TFA, respectively.

### 2.5. Changes in P. purpureum SCS-02 Phycobiliproteins Content under Different Nitrogen Concentrations

**Figure 5:**
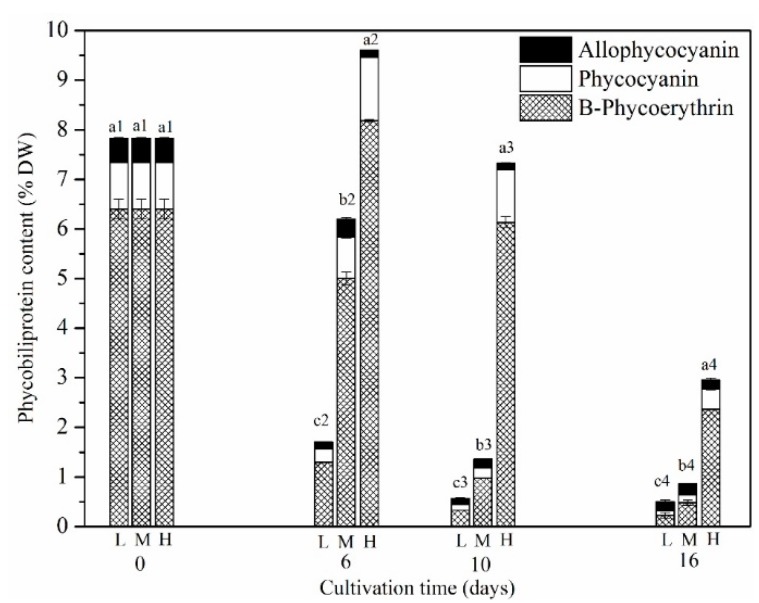
Changes of phycobiliproteins content of *P*. *purpureum* SCS-02 under different nitrogen concentrations. L: Low nitrogen supply (3.5 mM); M: medium nitrogen supply (5.9 mM); H: high nitrogen supply (17.6 mM); DW: dry weight. Different letters denoted significant differences among the values of phycoerythrin content in the different nitrogen concentration (a1–c1: day 0; a2–c2: day 6; a3–c3: day 10; a4–c4: day 16). The values shown are the averages of two biological replicates and three technical replicates ± standard deviation.

B-PE, R-phycocyanin (R-PC), allophycocyanin (APC) were the major phycobiliproteins in *P*. *purpureum* SCS-02. Our results showed that the proportion of B-PE reached 82% of the total phycobiliproteins; this was the primary reason for *P*. *purpureum* having a purple-red appearance (Figure 5 and Figure 1B). The proportion of R-PC and APC was 12% and 6%, respectively, of the total phycobiliproteins. Changes over time in the phycobiliproteins concentrations showed that the content of B-PE during the early stage of culture was higher than that during the middle and late stages. The nitrogen concentration had a notable effect on the B-PE, R-PC and APC content. For LN group and MN groups, the highest concentration of B-PE appeared on day 0, which was 6.41% DW. At the end of the cultivation period (day 16), the concentration of B-PE in these groups decreased to 0.22% DW and 0.48% DW, respectively. However, for the HN group, the highest content of B-PE was 8.18% DW, which appeared on day 6. On day 16, the concentration of B-PE decreased to 2.36% DW.

The ratio of B-PE to R-PC was more affected by the nitrogen concentration (Table 2). Under LN conditions, the decreasing ratio of B-PE to R-PC was faster than that shown under MN and HN conditions. Until the end of the culture period, the ratio of B-PE to R-PC was 2.25, 2.95 and 5.71 for the LN, MN and HN groups, respectively. These results suggest that nitrogen concentration has a considerable effect on B-PE content.

### 2.6. Effects of Different Nitrogen Concentrations on the Yields of B-PE, EPS and LC-PUFAs

The data in Table 3 show that the yields of B-PE, EPS and LC-PUFAs tended to increase with nitrogen and biomass concentration. As compared with the LN and MN groups, the HN group obtained a higher B-PE yield. On day 10, the B-PE yield of the HN group was the highest at 0.193 g L^−1^. As a function of time, the EPS yield gradually increased under the three nitrogen conditions. On day 16, the MN group obtained the highest EPS yield, which was 0.342 g L^−1^. Under HN conditions, the LC-PUFAs (ARA + EPA) yield was higher than that under the MN and LN conditions. On day 16, the LC-PUFAs (ARA + EPA) yield of the HN group reached 0.072 g L^−1^, which was 24.1% and 75.6% higher than the yield of the MN and LN groups, respectively.

## 3. Discussion

Nitrogen limitation or deficiency can significantly inhibit the growth of *Porphyridium* [9,12]. The present study showed that LN conditions led to an apparent reduction in the biomass concentration of *P*. *purpureum* SCS-02 (Figure 1A). This result is in agreement with those of many previous studies. For example, Hu et al. (2018) in their study reported that the biomass concentration of *P. cruentum* gy-h56 was improved by a maximum of 2.5-fold under conditions of nitrogen sufficiency as compared with nitrogen deprivation [9]. Results reported by Guihéneuf and Stengel (2015) showed that nitrogen deficiency caused a strong decrease in growth performance of *P*. *purpureum* PLY#539, likely because nitrogen is an essential component of many key enzymes in microalgae [7]. Moreover, nitrogen starvation could seriously decrease the photochemical efficiency and activity of reaction centers of *P*. *cruentum,* which contributed to reduced biomass concentration [24]. Interestingly, in the present study, the biomass concentration of the MN group was notably higher than that of the HN group between day 4 and day 8 (Figure 1A). We speculated that this exception might be due to differences in carbon distribution under different nitrogen concentrations. On day 4, nitrogen limitation had begun in the MN group, but not in the HN group (Figure 1C). Our previous research on *Chlorella vulgaris* showed that cells division were suppressed, and unit cell weight was rapidly increased due to the storage of energy and carbon under nitrogen-limited conditions, which could result in a great increase in biomass concentration. Although the cells were kept in normal division under nitrogen-sufficient conditions, the unit cell weight did not increase significantly [8]. In the present study, it was speculated that unit cell weight of MN of *P. purpureum* SCS-02 was higher than that of HN, but the cell density of MN was lower than that of HN between day 4 and day 8. Based on the following equations: biomass concentration (g L^−1^) = cell density (cell L^−1^) × unit cell weight (g cell^−1^), the above exceptional results were reasonably explained. Moreover, it could be inferred that cell density between day 4 and day 8 has less contribution to the biomass concentration in HN. In our future study, we will detect the change of cell density under different concentrations. With the consumption of nitrogen source, the HN group also entered into a state of nitrogen limitation. Due to the storage of energy and carbon for microalgal survival, the unit cell weight of *P. purpureum* SCS-02 in HN rapidly increased. Because of the high cell density of HN, it could achieve higher biomass concentrations than that of MN and LN. At the end of the cultivation period, the HN group reached up to 5.54 g L^−1^ of biomass concentration, which was far higher than concentrations reported in several prevous studies. The maximum biomass concentration in *P. cruentum* reported in the studies by Hu et al. (2018) and Razaghi et al. (2014) were 1.61 g L^−1^ and 1.22 g L^−1^, respectively [9,10]. This may be due to the constant supply of carbon available during the entire cultivation time in the present study.

Nitrogen limitation or deficiency can change carbon distribution towards carbohydrates or lipids instead of proteins [8]. In the present study, with the depletion of the nitrogen source, the carbohydrate content of *P*. *purpureum* SCS-02 significantly increased, but the protein content sharply declined (Figure 2A,C). This was consistent with the findings of the study by Fuentes-Grünewald et al. (2015), who reported that the carbohydrate content increased to 23% DW from 15% DW, and the protein content declined to 13% DW from 23% DW in batch culture with the depletion of nitrogen sources [23]. Rhodophyta polysaccharide (floridean starch) was shown to be the primary form of energy storage for *Porphyridium* [25]. Nitrogen limitation could accelerate the accumulation of polysaccharides in red algae. Razaghi et al. (2014) in their study observed that the carbohydrate content of *P. cruentum* at stationary phase was 0.4 g cell^−1^ in 1.6 of N:P ratio (nitrogen: phosphorus), but only 0.2 g cell^−1^ in 50 of N:P ratio [10]. For *P*. *purpureum* SCS-02, the total lipid content was similar to the total lipid contents reported by Becker et al. (1994) (9–14% DW) [26]. There was almost no change in the total lipid content of *P*. *purpureum* SCS-02 throughout the culture (9–12% DW) (Figure 2B). Cohen et al. (1990) in their study also did not observe any increase in the total lipid content during the stationary phase [27]. Coward et al. (2016) demonstrated that very little change occurred in the total lipid content under different light-emitting diodes [19]; the lipids present in red algae were primarily those of the functional membranes, and this content was relatively stable under normal culture conditions. Only when microalgae suffered stressful conditions were their membrane lipids (mainly the chloroplast membrane) decomposed. Collapse of the chloroplast membrane may cause cell apoptosis.

*P*. *purpureum* can secrete EPS, the average molecular weight of which reaches 1.4 × 10^6^ Da–1.7 × 10^6^ Da [3]. EPS is considered an optimal ingredient in cosmetics because of its excellent moisturising properties. In addition, EPS contains β-1,3-glycosidic bonds and sulphates, which endow EPS with anti-viral, antioxidant and radical-scavenging functions [2]. Our results showed that the concentration of EPS gradually increased with the culture time, but the accumulation rate of EPS revealed significant differences between different nitrogen concentrations (Figure 3). The LN group first entered nitrogen limitation on day 4. The accumulation rate of EPS under LN conditions was faster than that under MN and LN conditions between day 4 and day 8 (Figure 3), implying that LN conditions promote the accumulation of EPS in *P*. *purpureum* SCS-02. Arad et al. (1992) in their study reported that the highest polysaccharide contents per cell, including total cell-wall polysaccharide content and its distribution between bound and dissolved fractions, were obtained in nitrogen-deficient cultures of *Rhodella reticulate* [28]. After 6 days, the MN group also entered nitrogen-limiting conditions. But, interestingly, the accumulation rate of EPS in the MN group exceeded that of the LN and HN groups. The reasons for these results may be: (1) the LN group had lower photosynthetic carbon fixation than the MN group; and (2) the HN group was still growing under nitrogen sufficient conditions, and thus produced little EPS. The maximum concentration of EPS was 0.342 g L^−1^ in the present study. Coward et al. (2016) showed that *P. purpureum* CCAP 1380/3 excreted 2.05 g L^−1^ of EPS in batch culture by day 10 [19]. Sun et al. (2010) in their study demonstrated that the concentration of EPS produced by *P*. *cruentum* P.C-03, which was cultured semi-continuously in a flat plate photobioreactor, could reach up to 1.25 g L^−1^ by day 16 [11]. Therefore, to produce a high EPS yield, it was important to determine an optimal nitrogen concentration, which maintained not only the high photosynthetic efficiency of *P*. *purpureum*, but also created the stress of nitrogen limitation.

*P*. *purpureum* contains three types of phycobiliproteins: B-PE; PC; and APC [29]. Among these, B-PE is one of the most well-known pigment-protein complexes that is prized for its properties as a dye and an antioxidant, as well as its radical scavenging and antiallergic functions [30]. Nitrogen deficiency has a notable influence on the B-PE content of *Porphyridium* [12]. In the present study, the decreasing rate of B-PE under LN conditions was significantly higher than that under HN and MN conditions (Figure 5). The B-PE content under LN conditions was reduced by more than 90% on day 6. Guihéneuf et al. (2015) in their study showed that nitrogen starvation induced a strong decrease in PB content from 1.4% DW (day 9) to 0.6% DW (day 17) under nitrogen-limited conditions, and a more drastic decrease from 1.7% DW (day 0) to almost 0% DW (day 10) under nitrogen-starved conditions [12]. Dupré et al. (1994) reported that B-PE could serve as the nitrogen pool for microalgae [31]. Under nitrogen-deficient conditions, phycobiliproteins could be catabolised to supply amino acids for other important metabolism functions. The present study showed that the highest concentration of phycobiliproteins was 10.1% DW, the composition of which was 81.8% B-PE, 12.8% PC and 5.4% APC (Figure 5). The *P*. *purpureum* SCS-02 phycobiliproteins composition was similar to that revealed in a previous study by [22] (70% B-PE, 20% PC and 10% APC). In addition, LN caused a sharp decrease in the ratio of B-PE to PC (Table 2), which may indicate that, in *Porphyridium*, B-PE is be more sensitive to LN stress than is PC. In summary, nitrogen-sufficient conditions are the prerequisite for a high B-PE yield in *Porphyridium*. Fabregas et al. (1998) in their study reported that semi-continuous cultivation with 30% renewal rates notably increased the concentration of B-PE, suggesting that semi-continuous or continuous cultivation may be the most suitable conditions for B-PE production in *Porphyridium*.

*P*. *purpureum* can accumulate high ARA and EPA content [27]. ARA and EPA have important functions for the secretion of prostaglandin thromboxane and leukotriene, as well as infant brain development [32]. In the present study, EPA and ARA accounted for 28.38% TFA and 27.51% TFA in *P*. *purpureum* SCS-02, respectively (Figure 4). Kavitha et al. (2016) showed that the proportion of EPA and ARA reached 27.60% TFA and 29.9% TFA in *P*. *purpureum* through culture media optimisation [4]. The present study demonstrated that the proportion of EPA reached its maximum during the logarithmic phase of culture (day 6), perhaps because EPA was a major component of membrane lipids, made up primarily of glycolipids [13,27,33]. During the logarithmic phase, *P*. *purpureum* may synthesise a large amount of membrane lipids (mainly chloroplast membranes) to accommodate rapid cell growth. Furthermore, our results show that the proportion of EPA under HN conditions was higher than that under LN conditions, further supporting this hypothesis. Many previous studies reported that HN conditions contributed to the accumulation of membrane lipids [8,34]. Li et al. (2016) in their study reported that LN supply increased the proportion of neutral lipids, but reduced that of glycolipids and phospholipids in *C*. *vulgaris* JNU13 as compared with a HN supply [8]. Interestingly, it appears that the accumulation of ARA in response to nitrogen stress was not consistent across these studies. In the present study, the proportion of ARA in the LN group was 8.03% higher than was that of the HN group (Figure 4). Cohen (1990) in this study demonstrated that high ARA content was obtained under nitrogen-starvation conditions (2.9% DW) [27]. However, Hu et al. (2018) reported that nitrogen deficiency in *P. cruentum* gy-h56 caused a decrease in the proportion of ARA from 35.9% TFA under HN conditions to 28.2% TFA under LN conditions [9]. This may be due to differences between the strains of *Porphyridium*, differences in culture medium, and/or variations in the mode of cultivation. Nitrogen deficiency was reported to increase the accumulation of neutral lipids, but decrease LC-PUFAs. Cohen (1990) in this study suggested that the reason for this might be that the content of ARA increases in neutral lipids and in phospholipids, but not in glycolipids [27].

The present study showed that the maximum B-PE yield appeared in the HN group on day 10 (0.193 g L^−1^), the maximum EPS yield appeared in the MN group on day 16 (0.342 g L^−1^), and the maximum LC-PUFA yield appeared in the HN group on day 16 (0.072 g L^−1^) (Table 3). These results indicated that sufficient nitrogen was essential for the production of high B-PE and LC-PUFA yields. By calculation, the maximum productivity of B-PE, EPS and LC-PUFAs were 19.3 mg L^−1^ day^−1^, 21.6 mg L^−1^ day^−1^ and 4.5 mg L^−1^ day^−1^, respectively. Fabregas et al. (1998) in this study reported that, using semi-continuous cultivation with 30% renewal rates, the productivity of B-PE could be up to 18.3 mg L^−1^ day^−1^ [35]. Maximum EPA productivity was 5.29 mg L^−1^ day^−1^ at a renewal rate of 50%. At a renewal rate of 20%, the productivity of ARA was 6.91 mg L^−1^ day^−1^. This indicated that semi-continuous or continuous cultivation may be the most suitable method for the production of B-PE and LC-PUFAs in *Porphyridium*. In addition, Lutzu et al. (2017) exhibited a new method of culture called attached cultivation to promote the growth rate and EPS production of *P*. *cruentum*. The best EPS production under the attached cultivation reached 45% DW [36]. In the present study, it was found that LN conditions promoted the secretion of EPS. However, damage occurring to the photosystems during limited-nitrogen conditions negatively affected the secretion of EPS in *Porphyridium*. Therefore, based on the different accumulation characteristics of EPS, LC-PUFAs and B-PE, we here propose a two-step process. Firstly, *P*. *purpureum* would be cultured under HN conditions. When the logarithmic growth phase was reached, one-third of the algae cells would be harvested via centrifugation, allowing the *P*. *purpureum* biomass rich in LC-PUFAs and B-PE to be obtained. Secondly, one-third of the *P*. *purpureum* in the logarithmic growth phase would be used to inoculate the MN or LN mediums for the induction of EPS. The EPS-containing supernatant would then be subjected to the process of centrifugation, ultrafiltration for desalination and alcohol precipitation to obtain EPS. Finally, the remaining one-third of the original culture would be supplemented with HN medium for the next semi-continuous culture.

## 4. Materials and Methods

### 4.1. Microorganisms and Culture Conditions

Strain of *Porphyridium* sp. SCS-02 was isolated from the seawater samples, which were collected from the South China Sea by using repeated plate streaking. The 18S rRNA gene sequences and BLAST analysis revealed that the microalgal strain *Porphyridium* sp. SCS-02 was closely related to *Porphyridium purpureum* CCAP 1380-3 (99%) (Supplementary Figure S1). Thus, in the present study, *Porphyridium* sp. SCS-02 was named as *Porphyridium purpureum* SCS-02.

The stock culture was maintained in a 250-mL flask containing modified ASW medium (ASW). The starter culture was grown in a 6.0 cm × 60 cm glass column containing ASW (1200 mL) composed of: 462.0 mM NaCl; 26.8 mM MgSO_4_·7H_2_O; 27.5 mM MgCl_2_·7H_2_O; 10.2 mM CaCl_2_·2H_2_O; 17.6 mM NaNO_3_; 0.69 mM K_2_HPO_4_·3H_2_O; 0.48 mM NaHCO_3_; 11.7 μM EDTANa_2_·2H_2_O; 11.7 μM FeCl_3_·6H_2_O; 0.91 μM MnCl_2_·4H_2_O; 0.08 μM ZnSO_4_·7H_2_O; 0.02 μM Na_2_MoO_4_·2H_2_O; 0.04 μM Co(NO_3_)_2_·6H_2_O; 0.04 μM CuSO_4_·5H_2_O.

For the nitrogen treatment experiment, starter cultures in logarithmic phase were gently centrifuged (5000 rpm for 10 min) and washed with nitrogen-free ASW medium. The microalgal pellets were resuspended in a small volume of nitrogen-free ASW medium. The suspensions were then used to inoculate a 6.0 cm × 60 cm glass column photobioreactors containing ASW medium (1200 mL) with the following nitrogen concentrations: (1) 3.5 mM KNO_3_ (low level of nitrate supply, LN); (2) 5.9 mM KNO_3_ (medium level of nitrate supply, MN); and (3) 17.6 mM KNO_3_ (high level of nitrate supply, HN). The concentration of the starting biomass was 0.3 g L^−1^ (OD_750_ was 1.0). Illumination was provided by a bank of fluorescent lamps (Philips, T8, Koninklijke Philips Electronics N.V., Shanghai, made in China) on one side of the photobioreactor producing 350 μmol photons·m^−2^ s^−1^. The photoperiod was 24 h:0 h (light:dark), and the growth temperature was maintained at 25 ± 1 °C. The carbon source and agitation were supplied by bubbling CO_2_-enriched compressed air (1% CO_2_, *v:v*), which was filtered through the disposable sterile filter. Microalgal cells were harvested via centrifugation (8000 rpm for 10 min) for biochemical composition analysis on day 0, 6, 10 and 16. Microalgal growth was measured on day 0, 2, 4, 6, 8, 10, 12, 14 and 16. Each treatment had two biological replicates (*n* = 2), and each measurement had three technical replicates (*n* = 3). The biomass sludge was freeze-dried (FD-1-50, Beijing Boyikang Laboratory Instrument Co., Ltd., Beijing, China) and then stored at −20 °C.

### 4.2. Growth Measurement

Dry weight (DW) was measured by filtering a 10 mL aliquot of the culture through pre-weighed filters. After rinsing with ammonium bicarbonate, the filters were dried overnight at 80 °C, and reweighed.

### 4.3. Determination of Biochemical Composition

Freeze-dried biomass (100 mg DW) was used for the determination of biochemical composition, including total lipids, total carbohydrates and proteins. A modified Khozin-Goldberg’s method was employed to determine total lipid content [37]. Free lipid residual biomass was used to measure the amount of total carbohydrates and proteins. Ten mg DW of this biomass was hydrolysed with 1.0 N H_2_SO_4_ at 80 °C for 1 h. Total carbohydrate content was measured using the phenol-sulfuric acid method [38]. Free lipid residue (20 mg DW) was hydrolysed with 0.5 N NaOH at 80 °C for 20 min, and this process was repeated three times. Protein content was analysed colorimetrically using the Lowry method [39].

### 4.4. Phycobiliproteins Content

Freeze-dried biomass (20 mg DW) was used to determine the phycobiliproteins content. The phycobiliproteins were extracted with 0.1 M phosphate buffer (pH 6.8) using a freeze-thawing technique. The cells were extracted repeatedly until the algal residue had no obvious red colour, and the extracts were combined to a final volume of 50 mL. Absorbance of the extract was measured at 620 nm, 650 nm and 565 nm using a spectrophotometer (TU-1810, Persee Instrument Co., Ltd., Beijing, China). The contents of R-phycocyanin (R-PC), allophycocyanin (APC) and B-Phycoerythrin (B-PE) were estimated using the following equations [40]:

(1) $$\text{R-PC}\;(\mathrm{mg}\;{\mathrm{mL}}^{-1})=\frac{{\mathrm{OD}}_{620}-0.7\times {\mathrm{OD}}_{650}}{7.38}$$

(2) $$\mathrm{APC}\;(\mathrm{mg}\;{\mathrm{mL}}^{-1})=\frac{{\mathrm{OD}}_{650}-0.19\times {\mathrm{OD}}_{620}}{5.65}$$

(3) $$\text{B-PE}\;(\mathrm{mg}\;{\mathrm{mL}}^{-1})=\frac{{\mathrm{OD}}_{565}-2.8\times (R-\mathrm{PC})-1.34\times (\mathrm{APC})}{12.7}$$

R-PC (% DW) = R-PC (mg mL^−1^) × 50/20 (4)

APC (% DW) = APC (mg mL^−1^) × 50/20 (5)

B-PE (% DW) = B-PE (mg mL^−1^) × 50/20 (6)

Here, R-PC is the phycocyanin from red algae, APC is the allophycocyanin and B-PE is the phycoerythrin from the red algae.

### 4.5. Fatty Acid Composition

The freeze-dried biomass was transmethylated with 2% H_2_SO_4_ in a methanol: toluene mixture (90:10, *v/v*) at 80 °C for 1.5 h. FAMEs were analyzed using a gas chromatograph-mass spectrometer (GC-2014 with flame ionization detector, Shimadzu), equipped with a 100-m fused silica capillary column SP-2560. The temperature of the injection port was 260 °C. The column was temperature-programmed from 140 °C (with a hold of 5 min) to 240 ° C at 4 °C min^−1^ (with a hold of 20 min). The carrier gas was high-purity nitrogen, and the flow rate was 1.2 mL min^−1^. Individual peaks of FAMEs were identified by comparison of retention time with those of the authentic (37 species of fatty acid standards, NU-CHEK-PREP, INC, Elysian, MN, USA). Response factors of individual FAMEs were calculated by comparing with internal standard (methyl heptadecanoate, C17:0). The amount of individual FAMEs was calculated by the internal standard method.

Fatty acid content (% DW) = fatty acid weight (g)/algae power weight (g)(7)

Fatty acid yield (g L^−1^) = Fatty acid content (% DW) × biomass concentration (g L^−1^) (8)

### 4.6. EPS Concentration Determination

A 10 mL aliquot of the culture was centrifuged at 8000 rpm for 10 min. The supernatant was collected and stored at −20 °C for the determination of EPS concentration. In order to avoid the disturbance of monosaccharaides and oligosaccharides, the supernatant was dialyzed using a dialysis bag of 100–500 Da before measurement before the determination of EPS concentration. The EPS concentration was measured using the phenol-sulfuric acid method [38]. If the EPS concentration was too high, the supernatant was diluted to the proper concentration.

### 4.7. Calculation of the Yield of B-PE, EPS and LC-PUFAs

The yield of B-PE and LC-PUFAs was estimated using the following equations:

B-PE (g L^−1^) = DW (g L^−1^) × R-PC (% DW) (9)

LC-PUFAs (g L^−1^) = DW (g L^−1^) × ARA (% DW) + DW (g L^−1^) × EPA (% DW) (10)

### 4.8. Concentration of NO_3_^-^

10 mL of the culture was obtained and centrifuged at 8000 rpm for 10 min. The supernatant was collected and then filtered by using a piece of 0.22 µM filter membrane. The NO_3_^−^ concentration was determined by using an AutoAnalyzer3 (Bran-Luebbe, Norderstedt, Germany).

### 4.9. Statistical Analysis

All figures and tables showed the means and standard deviations of two independent biological replicates and three technical replicates. Statistical analysis (ANOVA) was performed using SPSS version 18.0 (SPSS Inc., Chicago, IL, USA) to confirm the differences between treatments. Multiple comparisons between different treatments of nitrogen concentration at the same cultivation time was performed using the S-N-K method (e.g., the content of lipids in HN versus that of MN on day 16; the content of lipids in MN versus that of LN on day 16; the content of lipids in HN versus that of LN on day 16). The difference between sample means of two neighbouring time point was analysed using the least significant difference (LSD) at α = 0.05. (e.g., the biomass concentration of HN on day 0 versus that of HN on day 2; the biomass concentration of HN on day 2 versus that of HN on day 4).

## 5. Conclusions

There are differences in the response of B-PE, LC-PUFAs and EPS to nitrogen concentration. Results of the present study indicated that sufficient nitrogen is essential for high yields of B-PE and LC-PUFAs, while LN conditions could promote the secretion of EPS. By calculation, the maximum productivity of B-PE, EPS and LC-PUFAs were 19.3 mg L^−1^ day^−1^, 21.6 mg L^−1^ day^−1^ and 4.5 mg L^−1^ day^−1^, respectively. The biomass concentration made a significant contribution to the final productivity of B-PE, EPS and LC-PUFAs. Therefore, a semi-continuous two-step approach was proposed to simultaneously produce *Porphyridium* biomass rich in B-PE, LC-PUFAs and EPS.

## Figures and Tables

**Table 1:** Yield of protein, total lipids and carbohydrate under different nitrogen concentrations.

-	* Protein (g L^−1^)	Total Lipids (g L^−1^)	Carbohydrates (g L^−1^)
3.5 mM KNO_3_	0.30 ± 0.02 ^c1^	0.33 ± 0.00 ^c2^	1.67 ± 0.00 ^c3^
5.9 mM KNO_3_	0.41 ± 0.09 ^b1^	0.44 ± 0.02 ^b2^	2.04 ± 0.02 ^b3^
17.6 mM KNO_3_	1.01 ± 0.01 ^a1^	0.63 ± 0.00 ^a2^	2.14 ± 0.08 ^a3^

* Different letters denoted significant differences among the values of the protein content, total lipids content and carbohydrate content on the different nitrogen concentration (a1–c1: protein; a2–c2: total lipids; a3–c3: carbohydrates). The values shown are the averages of two biological replicates and three technical replicates ± standard deviation.

**Table 2:** Ratio of B-PE to R-PC as a function of time of *Porphyridium purpureum* SCS-02.

-	* Day 0	Day 6	Day 10	Day 16
3.5 mM KNO_3_	6.75 ± 0.15 ^a1^	4.74 ± 0.12 ^c2^	2.87 ± 0.05 ^c3^	2.25 ± 0.06 ^a4^
5.9 mM KNO_3_	6.75 ± 0.15 ^a1^	6.01 ± 0.11 ^b2^	4.47 ± 0.04 ^b3^	2.95 ± 0.04 ^a4^
17.6 mM KNO_3_	6.75 ± 0.15 ^a1^	6.39 ± 0.05 ^a2^	5.76 ± 0.06 ^a3^	5.71 ± 0.02 ^a4^

* Different letters denoted significant differences among the values of ratio of B-PE to R-PC in the different nitrogen concentrations (a1–c1: day 0; a2–c2: day 6; a3–c3: day 10; a4–c4: day 16). The values shown are the averages of two biological replicates and three technical replicates ± standard deviation.

**Table 3:** Yield of B-PE, EPS and LC-PUFAs of *Porphyridium purpureum* SCS-02.

-	KNO_3_ Concentration	Day 0	Day 6	Day 10	Day 16
* B-PE (g L^−1^)	3.5 mM	0.019 ± 0.002	0.023 ± 0.002	0.008 ± 0.000 ^c1^	0.007 ± 0.000
	5.9 mM	0.019 ± 0.000	0.092 ± 0.004	0.030 ± 0.001 ^b1^	0.020 ± 0.001
	17.6 mM	0.018 ± 0.001	0.111 ± 0.002	**0.193 ± 0.002** ^a1^	0.131 ± 0.002
EPS (g L^−1^)	3.5 mM	0.025 ± 0.002	0.118 ± 0.002	0.191 ± 0.011	0.299 ± 0.023 ^b2^
	5.9 mM	0.029 ± 0.001	0.094 ± 0.002	0.206 ± 0.012	**0.342 ± 0.001** ^a2^
	17.6 mM	0.030 ± 0.002	0.094 ± 0.005	0.149 ± 0.002	0.289 ± 0.023 ^b2^
LC-PUFAs (ARA + EPA) (g L^−1^)	3.5 mM	0.005 ± 0.002	0.018 ± 0.002	0.026 ± 0.001	0.041 ± 0.001 ^c3^
	5.9 mM	0.005 ± 0.002	0.022 ± 0.001	0.039 ± 0.002	0.058 ± 0.000 ^b3^
	17.6 mM	0.005 ± 0.000	0.022 ± 0.001	0.044 ± 0.002	**0.072 ± 0.001** ^a3^

* Different letters denoted significant differences among the values of the protein content, total lipids content and carbohydrate content on the different nitrogen concentrations (a1–c1: B-PE yield; a2–c2: EPS yield; a3–c3: LC-PUFAs). The values shown are the averages of two biological replicates and three technical replicates ± standard deviation. B-PE: B-phycoerythrin; LC-PUFAs: long chain polyunsaturated fatty acids; EPS: exopolysaccharide; ARA: arachidonic acid (C20:4); EPA: eicosapentaenoic acid (C20:5).

## Supplementary Materials

The following are available online at http://www.mdpi.com/1660-3397/17/2/124/s1. Figure S1: Phylogenetic tree based on 18S rRNA gene sequence of *P*. *purpureum* SCS-02. The method of DNA extraction and PCR reaction were used as described by Li et al. 2016 [8]. The primer for the 18S rRNA gene sequence was A (5′-ACGCTTGTCTCAAAGATTA-3′) and B (5′-ACGGAAACCTTGTTACGA-3′). The 18S rRNA gene sequences were sequenced by BGI Tech Solutions Co., Ltd., Shenzhen, China.

## Abbreviations

The following abbreviations are used in this manuscript:

APC	allophycocyanin
ARA	arachidonic acid, C20:4
B-PE	B-phycoerythrin
DW	dry weight
EPS	exopolysaccharide
EPA	eicosapentaenoic acid, C20:5
HN	high nitrate supply
GLs	glycolipids
LC-PUFAs	long chain polyunsaturated fatty acids
LN	low nitrate supply
LSD	least significant difference
MN	medium nitrate supply
NLs	neutral lipids
OD_750_	optical density at the wavelength of 750 nm
PLs	phospholipids
R-PC	R-phycocyanin
S-N-K	Student-Newman-Keuls
TFA	total fatty acid

## References

[1] Patel A.K., Laroche C., Marcati A., Ursu A.V., Jubeau S., Marchal L., Petit E., Djelveh G., Michaud P. (2013). Separation and fractionation of exopolysaccharides from *Porphyridium cruentum*. Bioresour. Technol..

[2] Sun L., Wang C., Shi Q., Ma C. (2009). Preparation of different molecular weight polysaccharides from *Porphyridium cruentum* and their antioxidant activities. Int. J. Biol. Macromol..

[3] Bernaerts T.M.M., Kyomugasho C., Van Looveren N., Gheysen L., Foubert I., Hendrickx M.E., Van Loey A.M. (2018). Molecular and rheological characterization of different cell wall fractions of *Porphyridium cruentum*. Carbohydr. Polym..

[4] Kavitha M.D., Kathiresan S., Bhattacharya S., Sarada R. (2016). Culture media optimization of *Porphyridium purpureum*: Production potential of biomass, total lipids, arachidonic and eicosapentaenoic acid. J. Food Sci. Technol..

[5] Griffiths M.J., van Hille R.P., Harrison S.T.L. (2012). Lipid productivity, settling potential and fatty acid profile of 11 microalgal species grown under nitrogen replete and limited conditions. J. Appl. Phycol..

[6] Spolaore P., Joannis-Cassan C., Duran E., Isambert A. (2006). Commercial applications of microalgae. J. Biosci. Bioeng..

[7] Peccia J., Haznedaroglu B., Gutierrez J., Zimmerman J.B. (2013). Nitrogen supply is an important driver of sustainable microalgae biofuel production. Trends Biotechnol..

[8] Li T., Xu J., Gao B., Xiang W., Li A., Zhang C. (2016). Morphology, growth, biochemical composition and photosynthetic performance of *Chlorella vulgaris* (Trebouxiophyceae) under low and high nitrogen supplies. Algal Res..

[9] Hu H., Wang H.-F., Ma L.-L., Shen X.-F., Zeng R.J. (2018). Effects of nitrogen and phosphorous stress on the formation of high value LC-PUFAs in *Porphyridium cruentum*. Appl. Microbiol. Biot..

[10] Razaghi A., Godhe A., Albers E. (2014). Effects of nitrogen on growth and carbohydrate formation in *Porphyridium cruentum*. Cent. Eur. J. Biol..

[11] Sun L., Wang C., Ma C., Shi L. (2010). Optimization of renewal regime for improvement of polysaccharides production from *Porphyridium cruentum* by uniform design. Bioprocess Biosyst. Eng..

[12] Guiheneuf F., Stengel D.B. (2015). Towards the biorefinery concept: Interaction of light, temperature and nitrogen for optimizing the co-production of high-value compounds in *Porphyridium purpureum*. Algal Res..

[13] Khozin I., Adlerstein D., Bigongo C., Heimer Y.M., Cohen Z. (1997). Elucidation of the biosynthesis of eicosapentaenoic acid in the microalga *Porphyridium cruentum*. 2. Studies with radiolabeled precursors. Plant Physiol..

[14] Klyachkogurvich G.L., Doucha J., Kopezkii J., Ryabykh I.E., Semenenko V.E. (1994). Comparative investigation of fatty acid composition in lipids of various strains of *Porphyridium cruentum* and *Porphyridium aerugineum*. Russ. J. Plant Phys..

[15] Nuutila A.M., Aura A.M., Kiesvaara M., Kauppinen V. (1997). The effect of salinity, nitrate concentration, pH and temperature on eicosapentaenoic acid (EPA) production by the red unicellular alga *Porphyridium purpureum*. J. Biotechnol..

[16] Su G., Jiao K., Li Z., Guo X., Chang J., Ndikubwimana T., Sun Y., Zeng X., Lu Y., Lin L. (2016). Phosphate limitation promotes unsaturated fatty acids and arachidonic acid biosynthesis by microalgae *Porphyridium purpureum*. Bioprocess Biosyst. Eng..

[17] Del Pilar Sánchez-Saavedra M., Castro-Ochoa F.Y., Nava-Ruiz V.M., Ruiz-Güereca D.A., Villagómez-Aranda A.L., Siqueiros-Vargas F., Molina-Cárdenas C.A. (2018). Effects of nitrogen source and irradiance on *Porphyridium cruentum*. J. Appl. Phycol..

[18] Soanen N., Da Silva E., Gardarin C., Michaud P., Laroche C. (2016). Improvement of exopolysaccharide production by *Porphyridium marinum*. Bioresour. Technol..

[19] Coward T., Fuentes-Grunewald C., Silkina A., Oatley-Radcliffe D.L., Llewellyn G., Lovitt R.W. (2016). Utilising light-emitting diodes of specific narrow wavelengths for the optimization and co-production of multiple high-value compounds in *Porphyridium purpureum*. Bioresour. Technol..

[20] Rogova N., Springer M., Tsoglin L., Franke H., Pulz O. (1996). The influence of low and high oxygen concentration on the yield and spectrum of fatty acids in *Porphyridium cruentum*. J. Plant Physiol..

[21] Singh S., Arad S., Richmond A. (2000). Extracellular polysaccharide production in outdoor mass cultures of *Porphyridium* sp. in flat plate glass reactors. J. Appl. Phycol..

[22] Kathiresan S., Sarada R., Hattacharya S., Ravishankar G.A. (2007). Culture media optimization for growth and phycoerythrin production from *Porphyridium purpureum*. Biotechchnol. Bioeng..

[23] Fuentes-Gruenewald C., Bayliss C., Zanain M., Pooley C., Scolamacchia M., Silkina A. (2015). Evaluation of batch and semi-continuous culture of *Porphyridium purpureum* in a photobioreactor in high latitudes using Fourier Transform Infrared spectroscopy for monitoring biomass composition and metabolites production. Bioresour. Technol..

[24] Zhao L.-S., Li K., Wang Q.-M., Song X.-Y., Su H.-N., Xie B.-B., Zhang X.-Y., Huang F., Chen X.-L., Zhou B.-C. (2017). Nitrogen Starvation Impacts the Photosynthetic Performance of *Porphyridium cruentum* as Revealed by Chlorophyll a Fluorescence. Sci. Rep..

[25] Li S.Y., Lellouche J.P., Shabtai Y., Arad S. (2001). Fixed carbon partitioning in the red microalga *Porphyridium* sp. (Rhodophyta). J. Phycol..

[26] Becker (1994). Microalgae—Biotechnology and Microbiology.

[27] Cohen Z. (1990). The production potential of eicosapentaenoic and arachidonid acids by the red alga *Porphyridium Cruentum*. J. Am. Oil Chem. Soc..

[28] Arad S.M., Lerental Y.B., Dubinsky O. (1992). Effect of nitrate and sulfate starvation on polysaccharide formation in *Rhodella Reticulata*. Bioresour. Technol..

[29] Ma S.Y., Wang G.C., Sun H.B., Zeng C.K. (2003). Characterization of the artificially covalent conjugate of B-phycoerythrin and R-phycocyanin and the phycobilisome from *Porphyridium cruentum*. Plant Sci..

[30] Sekar S., Chandramohan M. (2008). Phycobiliproteins as a commodity: Trends in applied research, patents and commercialization. J. Appl. Phycol..

[31] Dupre C., Guary J.C., Grizeau D. (1994). Effect of photon fluence rate, nitrogen limitation and nitrogen recovery on the level of phycoerythrin in the unicellular alga, *Rhodosorus marinus* (Rhodophyceae). Physiol. Plant..

[32] Ward O.P., Singh A. (2005). Omega-3/6 fatty acids: Alternative sources of production. Process Biochem..

[33] Naumann I., Darsow K.H., Walter C., Lange H.A., Buchholz R. (2007). Identification of sulfoglycolipids from the alga *Porphyridium purpureum* by matrix-assisted laser desorption/ionisation quadrupole ion trap time-of-flight mass spectrometry. Rapid Commun. Mass Spectrom..

[34] Msanne J., Xu D., Konda A.R., Casas-Mollano J.A., Awada T., Cahoon E.B., Cerutti H. (2012). Metabolic and gene expression changes triggered by nitrogen deprivation in the photoautotrophically grown microalgae *Chlamydornonas reinhardtii* and *Coccomyxa* sp C-169. Phytochemistry.

[35] Fabregas J., Garcia D., Morales E., Dominguez A., Otero A. (1998). Renewal rate of semicontinuous cultures of the microalga *Porphyridium cruentum* modifies phycoerythrin, exopolysaccharide and fatty acid productivity. J. Ferment. Bioeng..

[36] Lutzu G.A., Zhang L., Zhang Z., Liu T. (2017). Feasibility of attached cultivation for polysaccharides production by *Porphyridium cruentum*. Bioprocess Biosyst. Eng..

[37] Khozin-Goldberg I., Shrestha P., Cohen Z. (2005). Mobilization of arachidonyl moieties from triacylglycerols into chloroplastic lipids following recovery from nitrogen starvation of the microalga *Parietochloris incisa*. BBA Mol. Cell Biol. Lipids.

[38] Dubois M., Gilles K.A., Hamilton J.K., Rebers P.A., Smith F. (1956). Colorimetric method for determination of sugars and related substances. Anal. Chem..

[39] Lowry O.H., Rosebrough N.J., Farr A.L., Randall R.J. (1951). Protein measurement with Folin-phenol reagent. J. Biol. Chem..

[40] De Marsac N.T., Houmard J. (1988). Complementary chromatic adaptation: Physiological conditions and action spectra. Method Enzym..

