# Supplementary material for: Growth and Biochemical Composition of Porphyridium purpureum SCS-02 under Different Nitrogen Concentrations
Source: Mar Drugs. 2019 Feb 20;17(2):124. doi: 10.3390/md17020124 (PMC6410139; doi:10.3390/md17020124)
Supplement: Supplementary file 1 [file marinedrugs-17-00124-s001.pdf]

Supplementary Materials

Growth and Biochemical Composition of *Porphyridium purpureum* SCS-02 under Different Nitrogen Concentrations

Tao Li [1], Jin Xu [2], Houbo Wu [1], Peiliang Jiang [2], Zishuo Chen [1] and Wenzhou Xiang [1,*]

[1] CAS Key Laboratory of Tropical Marine Bio-resources and Ecology, Guangdong Key Laboratory of Marine Materia Medica, Institution of South China Sea Ecology and Environmental Engineering, RNAM Center for Marine Microbiology, South China Sea Institute of Oceanology, Chinese Academy of Sciences, Guangzhou 510301, China; taoli@scsio.ac.cn (T.L.); wuhoubo@scsio.ac.cn (H.W.); 18390943716@163.com (Z.C.)

[2] Key Laboratory of Renewable Energy, Guangzhou Institute of Energy Conversion, Chinese Academy of Sciences, Guangzhou 510640, China; xujin@ms.giec.ac.cn (J.X.); jiangyang9977@163.com (P.J.)

* Correspondence: xwz@scsio.ac.cn; Tel.: +86-020-8902-3223

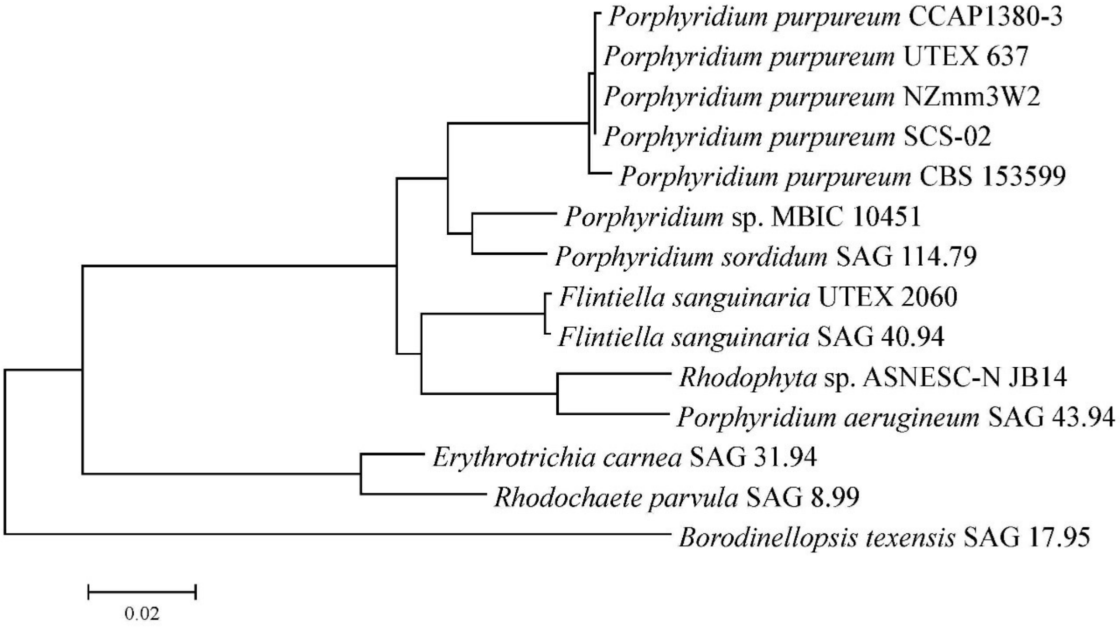

Figure S1 Phylogenetic tree based on 18S rRNA gene sequence of *P. purpureum* SCS-02.The method of DNA extraction and PCR reaction were used as described by Li et al. 2016 [8]. The primer for the 18S rRNA gene sequence was A (5′-ACGCTTGTCTCAAAGATTA-3′) and B (5′-ACGGAAACCTTGTTACGA-3′). The 18S rRNA gene sequences were sequenced by BGI Tech Solutions Co., Ltd. Shenzhen, China.
